# Anti-inflammatory activities of *Aedes aegypti* cecropins and their protection against murine endotoxin shock

**DOI:** 10.1186/s13071-018-3000-8

**Published:** 2018-08-14

**Authors:** Lin Wei, Yang Yang, Yandong Zhou, Min Li, Hailong Yang, Lixian Mu, Qian Qian, Jing Wu, Wei Xu

**Affiliations:** 1 Jiangsu Key Laboratory of Infection and Immunity, Institutes of Biology and Medical Sciences, Soochow University, Suzhou, 215123 Jiangsu China; 2 School of Basic Medical Sciences, Kunming Medical University, Kunming, 650500 Yunnan China

**Keywords:** Mosquito, *Aedes aegypti*, Cecropin, Anti-inflammation

## Abstract

**Background:**

Mosquitoes are armed with physiologically active compounds to suppress the host immunity including host inflammatory reaction. However, the specific anti-inflammatory components in mosquitoes remain unknown.

**Results:**

By searching for the immunomodulatory molecules from the mosquito *Aedes aegypti* (Diptera: Culicidae) at NCBI for anti-inflammatory function, five cecropins (for short in this study: *Aeae*Cec1, 2, 3, 4 and 5) were selected. *Aeae*Cec1-5 efficiently inhibited the expression of inducible nitric oxide synthase (iNOS), nitrite, tumor necrosis factor-α (TNF-α), interleukin-1β (IL-1β) and interleukin-6 (IL-6) in lipopolysaccharide (LPS)-stimulated mouse peritoneal macrophages and human peripheral blood mononuclear cells (PBMCs) with low toxicity to mammalian cells. Among the five analogues, *Aeae*Cec5 had the strongest anti-inflammatory activity, and generated an additive effect with other *Aeae*Cec peptides. In a mouse model of endotoxin shock, *Aeae*Cec1-5 effectively reduced TNF-α, IL-1β and IL-6 expression in lungs, serum and peritoneal lavage and correspondingly reduced lung damage and edema, with *Aeae*Cec5 showing the best protection. In mice infected with *Escherichia coli* or *Pseudomonas aeruginosa*, administration of *Aeae*Cec5 reduced the production of TNF-α, IL-1β and IL-6 and correspondingly reduced lung tissue damage. These effects of *Ae. aegypti Aeae*Cec1-5 were attributed to an efficient inhibition of the activation of mitogen-activated protein kinases (MAPKs) and transcriptional nuclear factor-κB (NF-κB) signaling pathways, as well as partial neutralization of LPS.

**Conclusions:**

The current work characterized the specific anti-inflammatory agents in *Ae. aegypti* and provided *Aeae*Cec5 as a potent anti-endotoxin peptide that could serve as the basis for the development of anti-inflammatory therapy.

**Electronic supplementary material:**

The online version of this article (10.1186/s13071-018-3000-8) contains supplementary material, which is available to authorized users.

## Background

Hematophagous arthropods like mosquito vectors are armed with a diverse group of active compounds with angiogenic, anticoagulant, vasodilatory and immunomodulatory properties, which facilitate adult female arthropods to finish blood meal acquisition and maintain pathogens before their transmission during blood-feeding [1–11]. An investigation of the crude salivary gland homogenates of *Anopheles albimanus* showed that the crude homogenates could oxidize noradrenalin and effectively inhibit vasoconstrictive pathways, thus promoting successful mosquito feeding [12]. Apyrases, the specific components in saliva of anopheline and culicine mosquitoes, were shown to inhibit ADP-induced platelet aggregation and limit local blood coagulation for successful blood-feeding [5, 13]. Two novel neuropeptides named sialokinin-I and II, identified from the salivary gland of mosquito *Ae. aegypti*, shared amino acid homology with mammalian substance P and had smooth muscle contracting activity [11]. Mosquito tachykinins are highly conserved neuropeptides among anopheline mosquitoes (*Anopheles gambiae*) and culicine mosquitoes (*Aedes triseriatus*) [14].

Apart from the above anticoagulant compounds, mosquitoes are equipped with immunomodulatory molecules involved in interference with host immunity, such as antimicrobial peptides (AMPs), immunosuppressors, amongst others [15, 16]. Mosquito AMPs were initially characterized for their antimicrobial activity *in vitro* [16–18]. Defensins, gambicins and cecropins comprise the three major AMP families in mosquitoes [15, 16, 19]. Mosquito defensins possess six conserved cysteine residues which form three intramolecular disulfide bonds linked in the Cys1-Cys4, Cys2-Cys5 and Cys3-Cys6 pattern [15]. Gambicins have eight cysteine residues which form four intramolecular disulfide bonds linked in the Cys1-Cys3, Cys2-Cys4, Cys5-Cys7 and Cys6-Cys8 pattern [16]. Cecropins are linear peptides lacking cysteine residues and form both amidated and nonamidated isoforms with a low molecular weight of about 4 kDa [20]. *In vitro* analyses of their antimicrobial activities indicated that they possessed different antimicrobial spectra. Mosquito defensins are primarily active to Gram-positive bacteria, while gambicins and cecropins are primarily active to both Gram-positive and Gram-negative bacteria [15]. Previous work indicated that *Culex pipiens* and *Ae. aegypti* feeding could modulate the cytokine production in C3H/HeJ mice [21]. Although the crude saliva or salivary gland extracts of mosquitoes showed serial immunomodulatory activities [8, 22, 23], active component composition in mosquitoes and its anti-inflammatory activity needs extensive study.

AMPs play key roles in the innate immune responses of vertebrates and invertebrates. AMPs were initially known as bactericidal peptides, and recently have become promising candidates of immunomodulatory peptides [15, 24–29]. In vertebrates, cathelicidins constitute a large group of AMPs with various immunomodulatory functions [28, 30–32], including anti-inflammatory activity [26, 33, 34]. In invertebrates, cecropins comprise one of the major AMP families with immunomodulatory activities focused on anti-inflammation [35]. Three cecropins (cecropin A, papiliocin and cecropin-A2) derived from non-bloodsucking insects *Hyalophora cecropia* [27], *Papilio xuthus* [36] and *Musca domestica* [37] with potent anti-inflammatory activities were characterized. Two cecropins (cecropin-TY1 and *Siba*cec) derived from blood-feeding insects *Tabanus yao* [25] and *Simulium bannaense* [7] were characterized as promising anti-inflammatory agents that possibly suppressed host inflammatory reaction during feeding. These naturally occurring cecropins with anti-inflammatory activities were only determined *in vitro*. In mosquitoes, cecropins are a major family of inducible AMPs that identified for anti-microbe *in vitro*, and a total of five cecropins have been identified in *Ae. Aegypti* [17, 38–40]. Mosquitoes are known as major vectors of numerous infective pathogens. However, no anti-inflammatory effects of cecropin peptides have been characterized in mosquitoes to date.

As described in our previous papers, cecropin-TY1 [25] and *Siba*cec [7] were characterized for the specific anti-inflammatory agents for the blood-feeding insects *T. yao* and *S. bannaense*. To identify the specific anti-inflammatory agents in the medically important blood-feeding mosquito vector *Ae. aegypti*, we searched the immune-related molecules from the genome sequences of *Ae. aegypti* at the National Center for Biotechnology Information (NCBI) [41, 42], and a total of five cecropins have been identified. To understand whether *Ae. aegypti* cecropins possess anti-inflammatory activities like cecropin-TY1 and *Siba*cec derived from blood-feeding insects *T. yao* [25] and *S. bannaense* [7], respectively, these five cecropins were selected as anti-inflammatory peptide candidates. We assessed their cytotoxicity against mammalian cells. We examined the inhibitory effects of *Aeae*Cec1-5 on iNOS, nitrite, TNF-α, IL-1β and IL-6 expression in murine peritoneal macrophages and human PBMCs. We also investigated the underlying mechanism by detecting whether *Aeae*Cec1-5 suppressed MAPKs and NF-κB signaling activation, as well as neutralized LPS. We further explored the anti-inflammatory activities of *Aeae*Cec1-5 in a murine endotoxic shock model and the protective roles of *Aeae*Cec5 in Gram-negative bacteria-infected mice. Our data characterize the anti-inflammatory components in *Ae. aegypti* and indicate the value of the *Ae. aegypti* cecropins as a basis for future development of anti-inflammatory agents.

## Methods

### Mice, bacteria and peptides

C57BL/6 mice (female, 18–20 g) were purchased from Shanghai Slac Animal Co. Inc. and housed in a pathogen-free facility. Gram-negative bacteria including *E. coli* (ATCC 25922) and *P. aeruginosa* (ATCC 27853) were cultured in Luria-Bertani broth at 37 °C.

The amino acid sequence and related information of the five *Ae. aegypti* cecropins are listed in Additional file 1: Table S1. All of the peptides in this study were synthesized by GL Biochem (Shanghai) Ltd. (Shanghai, China). The synthetic peptides were subjected to an automated Edman degradation protein sequencer and MALDI-TOF mass spectrometry to confirm the accuracy of amino acid sequence and the purity (> 98%), respectively.

### Mammalian cell culture

Mouse peritoneal macrophages from C57BL/6 mice were collected as previously described [25, 43]. Cells were cultured in RPMI-1640 supplemented with antibiotics (100 U/ml penicillin, 100 μg/ml streptomycin, Gibco, California, USA) and 10% fetal bovine serum. Human PBMCs were isolated according to a previous method [31]. Briefly, venous blood from healthy volunteers was collected into heparin-containing Vacutainer tubes (BD Biosciences, New Jersey, USA), diluted with an equal volume of PBS (pH 7.4) and separated by density gradient centrifugation using Lympholyte-poly cell separation media (Cedarlane, Ontario, Canada). Mononuclear cell layers were collected, washed and cultured in RPMI 1640 with 10% FBS, 2 mM L-glutamine, and 1 mM sodium pyruvate (Invitrogen, California, USA). Vero E6 cells were gifted by Dr Chunsheng Dong and cultured in DMEM supplemented with antibiotics and 10% FBS. All cells were cultured in a humidified incubator under 5% CO_2_ at 37 °C.

### Cytotoxic assay

Cell Counting Kit-8 (CCK-8) was used to detect the cytotoxicity of *Aeae*Cec1-5 against mammalian cells. In brief, mouse peritoneal macrophages, human PBMCs and Vero E6 cells were seeded in 96-well plates (2×10^4^ cells/well, 100 μl/well). After cells adhered to culture plate, the culture medium was transferred with a two-fold dilution series of *Aeae*Cec1-5 beginning with 50 μM (100 μl) prepared in serum-free RPMI-1640 for mouse macrophages and human PBMCs, and serum-free DMEM for Vero E6 cells. After incubation at 37 °C for 24 h, CCK-8 solution (10 μl/well) was added for an additional 4 h incubation. The absorbance at 450 nm was recorded on a microplate reader (Epoch Etock, BioTek, Vermont, USA).

### Nitric oxide assay

Mouse peritoneal macrophages or human PBMCs in 24-well plates (2.5×10^5^ cells/well) were incubated at 37 °C with LPS (100 ng/ml, from *E. coli* 0111:B4; Sigma-Aldrich, Missouri, USA) in the presence or absence of *Aeae*Cec1-5 (5 μM). Cells were harvested for determination of the transcription level of iNOS by qPCR after incubation for 6 h [33, 34]. After incubation for 24 h, the culture supernatant was collected, mixed with an equal volume of Griess reagent (Beyotime, Jiangsu, China), and incubated for 10 min at room temperature. NO production (nitrite accumulation) was measured on a microplate reader at 540 nm [26].

### ELISA assay of cytokines

The expression levels of pro-inflammatory cytokines were evaluated after the addition of 100 ng/ml of LPS (from *E. coli* 0111:B4; Sigma-Aldrich) to mouse peritoneal macrophages or human PBMCs in 24-well plates (2.5×10^5^ cells/well) in the presence or absence of *Aeae*Cec1-5 (5 μM). The cells were incubated for 6 h at 37°C. TNF-α, IL-1β and IL-6 levels in the supernatant were measured using mouse or human cytokine ELISA kits (eBioscience, California, USA) according to the instructions.

### qPCR

Total RNA from cells and tissues were extracted using Trizol reagent (Life Tech, California, USA). cDNA was synthesized using PrimeScript® reverse transcriptase kit (Takara, Dalian, China). SYBR green master mix (Takara) was used for a two-step qPCR assay on a Realplex Mastercycler real-time PCR system (Eppendorf, Hamburg, Germany) according to the manufacturer’s instructions. Transcription levels of target genes were normalized to GAPDH and calculated by the ΔΔCt method. The accuracy of qPCR results was verified by melting curve analysis. Primers used in the qPCR assay are listed in Additional file 1: Table S2.

### Western blot analysis

Mouse peritoneal macrophages in 6-well plates (2.5×10^6^ cells/well) were stimulated with LPS (100 ng/ml, from *E. coli* 0111:B4; Sigma-Aldrich) in the presence or absence of *Aeae*Cec1-5 (1.25, 2.5 and 5 μM). After treatment for 30 min, macrophages were harvested and lysed with RIPA lysis buffer (Beyotime). Total protein (40 μg) was separated by SDS-PAGE, and transferred to a polyvinylidene difluoride membrane. Then the membrane was blocked by incubation with 5% BSA (BD Biosciences) in Tris-buffered solution Tween-20. The signals were measured with primary antibodies (1:2000, Cell Signaling Technology, Massachusetts, USA) overnight at 4 °C, secondary antibody (1:5000, Cell Signaling Technology) for 1 h at room temperature using an enhanced chemiluminescence kit (Tiangen Biotech, Beijing, China) [26].

### LPS neutralization assay

LPS neutralization properties of peptides were measured using a ToxinSensor™ chromogenic LAL endotoxin assay kit (GenScript, Nanjing, China) according to the kit instructions [34]. Briefly, a two-fold dilution series of peptides (20 μl/well) and an equal volume of *E. coli* LPS solution (5 endotoxin units/ml) provided by the kit were mixed in a pyrogen-free 96-well plate. LAL water (20 μl/well) and an equal volume of LPS were mixed as a blank control. After incubation at 37 °C for 30 min, LAL reagent (20 μl/well) was added and incubated at 37 °C for 10 min. Then chromogenic substrate (40 μl/well) reagent was added and incubated at 37 °C for another 6 min. Finally, color stabilizers 1, 2 and 3 (40 μl/well) were added and the absorbance at 545 nm was monitored on a microplate reader (Epoch Etock, BioTek). LPS-neutralizing activity was calculated as (A_*blank*_ -A_*peptide*_ )/A_*blank*_ × 100%.

### *In vivo* anti-inflammatory effect in LPS-challenged mice

C57BL/6 mice (female, 18–20 g, *n*=10) were intraperitoneally injected with 10 mg/kg LPS (from *E. coli* 0111:B4; Sigma-Aldrich). Individual *Aeae*Cec1-5 (10 mg/kg), co-administration of *Aeae*Cec1-5 (2 mg/kg each) or an equal volume of vehicle (PBS) was intraperitoneally injected into the mice 30 min after LPS injection. Mice were sacrificed 4 h after treatment. Blood, peritoneal lavage and lungs were collected. For lung edema evaluation, the wet weight was taken, and the lungs were dried in an oven at 80°C for 48 h until achieving stable dry weight [44].

### *In vivo* protective activity of *Aeae*Cec5 against Gram-negative bacteria infection

C57BL/6 mice (female, 18–20 g, *n*=10) were intraperitoneally challenged with two major infective bacteria in clinical infectious diseases, *E. coli* or *P. aeruginosa* (2×10^7^ CFUs/mouse). *Aeae*Cec5 (10 mg/kg) or PBS was intraperitoneally administered into mice after bacterial infection. Blood, peritoneal lavage and tissues were collected 18 h post *Aeae*Cec5 administration [44].

### Histopathological assay

Tissues were collected and fixed in 10% formalin solution for 24 h. After dehydration by an increasing concentration of alcohol, tissues were embedded in paraffin and sectioned into a thickness of 5 μm section using a Histocut (Leica, Solms, Germany). Sections were stained with hematoxylin and eosin (H&E), and observed by light microscopy (Nikon Eclipse TE2000-S, Tokyo, Japan).

### Statistical analysis

GraphPad Prism 5.0 (GraphPad Software Inc., San Diego, CA, USA) was used to perform statistical analysis. Data are presented as mean ± SEM, and compared using two-tailed equal variance Student’s t-test. Values corresponding to *P*<0.05 were considered statistically significant.

## Results

### *Aeae*Cec1-5 inhibited LPS-induced NO production in mouse macrophages and human PBMCs

**Fig. 1:**
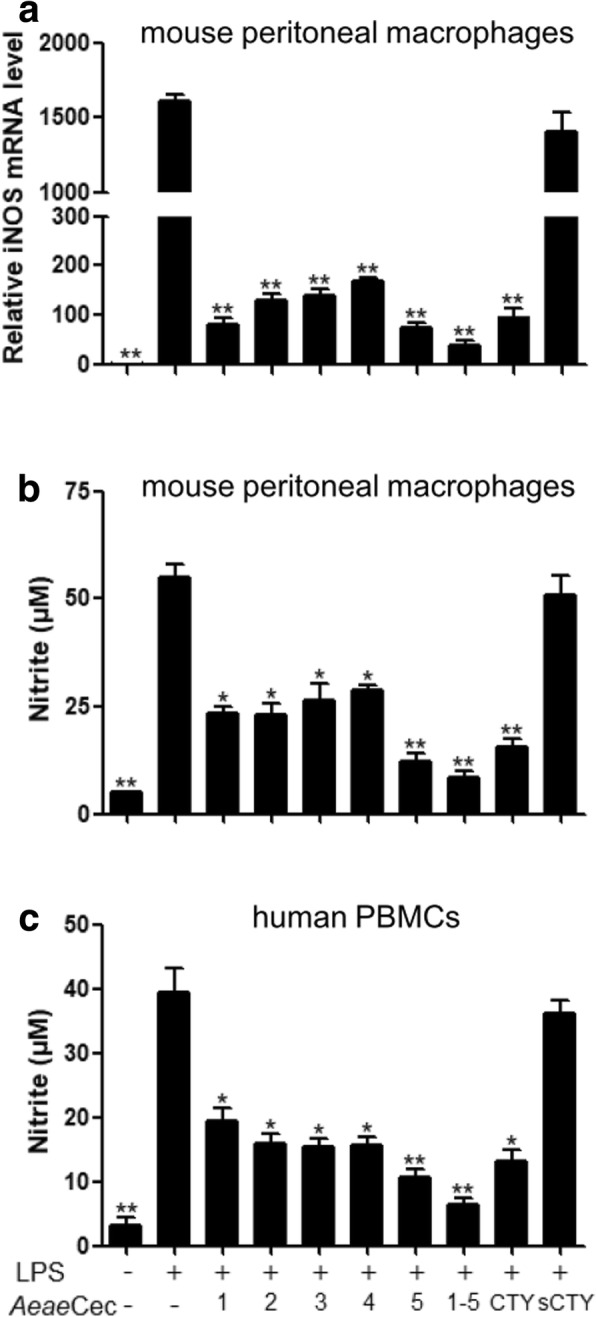
Inhibitory effects of *Ae. aegypti* cecropins on LPS-stimulated NO production in mouse peritoneal macrophages and human PBMCs. Mouse peritoneal macrophages or human PBMCs were incubated at 37 °C with *E. coli* LPS (100 ng/ml) in the presence or absence of 5 μM of each *Aeae*Cec peptides or a mixture of five peptides (1 μM each) or vehicle (PBS) or 5 μM of cecropin-TY1 (CTY) and scrambled cecropin-TY1 (sCTY) as control peptide [25]. **a** Cells were harvested for determination of the transcription level of iNOS by qPCR after incubation for 6 h. **b**, **c** After incubation for 24 h, the culture supernatant of mouse peritoneal macrophages (**b**) or human PBMCs (**c**) were detected for the NO production (nitrite accumulation) using Griess reagent. Data are presented as mean ± SEM from three independent experiments. *P < 0.05, **P < 0.01

In order to identify anti-inflammatory molecules in mosquitoes, five cecropins of *Ae. aegypti* were selected for anti-inflammatory assay *in vitro*. First, the effects of *Aeae*Cec1-5 on LPS-induced NO production in mouse peritoneal macrophages were determined, including iNOS transcription and nitrite production. As illustrated in Fig. 1a, LPS significantly induced iNOS transcription, while all *Aeae*Cec peptides (at 5 μM) markedly blocked iNOS transcription (reduction by more than 90% compared to LPS-induced level, *Aeae*Cec1: *t*_(4)_ = 12.75, *P* = 0.0002; *Aeae*Cec2: *t*_(4 )_= 12.23, *P* = 0.0003; *Aeae*Cec3: *t*_(4)_ = 12.20, *P* = 0.0003; *Aeae*Cec4: *t*_(4)_ = 11.99, *P* = 0.0003; *Aeae*Cec5: *t*_(4)_ = 12.79, *P* = 0.0002) with *Aeae*Cec5 as the most efficient (95.3%). As a result, LPS stimulation induced nitrite accumulation up to 55.2 μM in the culture medium, and all *Aeae*Cec peptides significantly inhibited this nitrite accumulation effect (*Aeae*Cec1: *t*_(4)_ = 11.02, *P* = 0.0004; *Aeae*Cec2: *t*_(4)_ = 10.11, *P* = 0.0005; *Aeae*Cec3: *t*_(4)_ = 7.219, *P* = 0.002; *Aeae*Cec4: *t*_(4)_ = 10.13, *P* = 0.0005; *Aeae*Cec5: *t*_(4)_ = 14.08, *P* = 0.0001; Fig. 1b). 5 μM of *Aeae*Cec1-4 reduced the nitrite level to 23.2–28.7μM, while *Aeae*Cec5 displayed the best inhibitory activity (12.1 μM). Consistent with that, in human PBMCs, *Aeae*Cec1-5 markedly reduced LPS-induced nitrite accumulation (*Aeae*Cec1: *t*_(4)_ = 5.981, *P* = 0.0039; *Aeae*Cec2: *t*_(4)_ = 7.41, *P* = 0.0018; *Aeae*Cec3: *t*_(4)_ = 8.063, *P* = 0.0013; *Aeae*Cec4: *t*_(4)_ = 7.669, *P* = 0.0016; *Aeae*Cec5: *t*_(4)_ = 9.402, *P* = 0.0007; Fig. 1c) with *Aeae*Cec5 as the most efficient (nitrite, 39.4 μM reduced to 10.7 μM, 72.8% reduction). To see if *Aeae*Cec1-5 had interactive effects on the inhibition of LPS-induced NO production, *Aeae*Cec1-5 mixtures (1 μM each) were added to the culture. In the mouse peritoneal macrophages, *Aeae*Cec1-5 mixtures inhibited 97.4% LPS-induced iNOS transcription and 84.2% LPS-induced nitrite accumulation. A similar result was seen in LPS-stimulated human PBMCs. These data indicated that *Aeae*Cec1-5 could potently inhibit LPS-induced NO production in macrophages and monocytes, and co-administration of *Aeae*Cec1-5 showed the best inhibitory effect (Fig. 1a-c).

### *Aeae*Cec1-5 inhibited LPS-induced pro-inflammatory cytokines production in mouse macrophages and human PBMCs

**Fig. 2:**
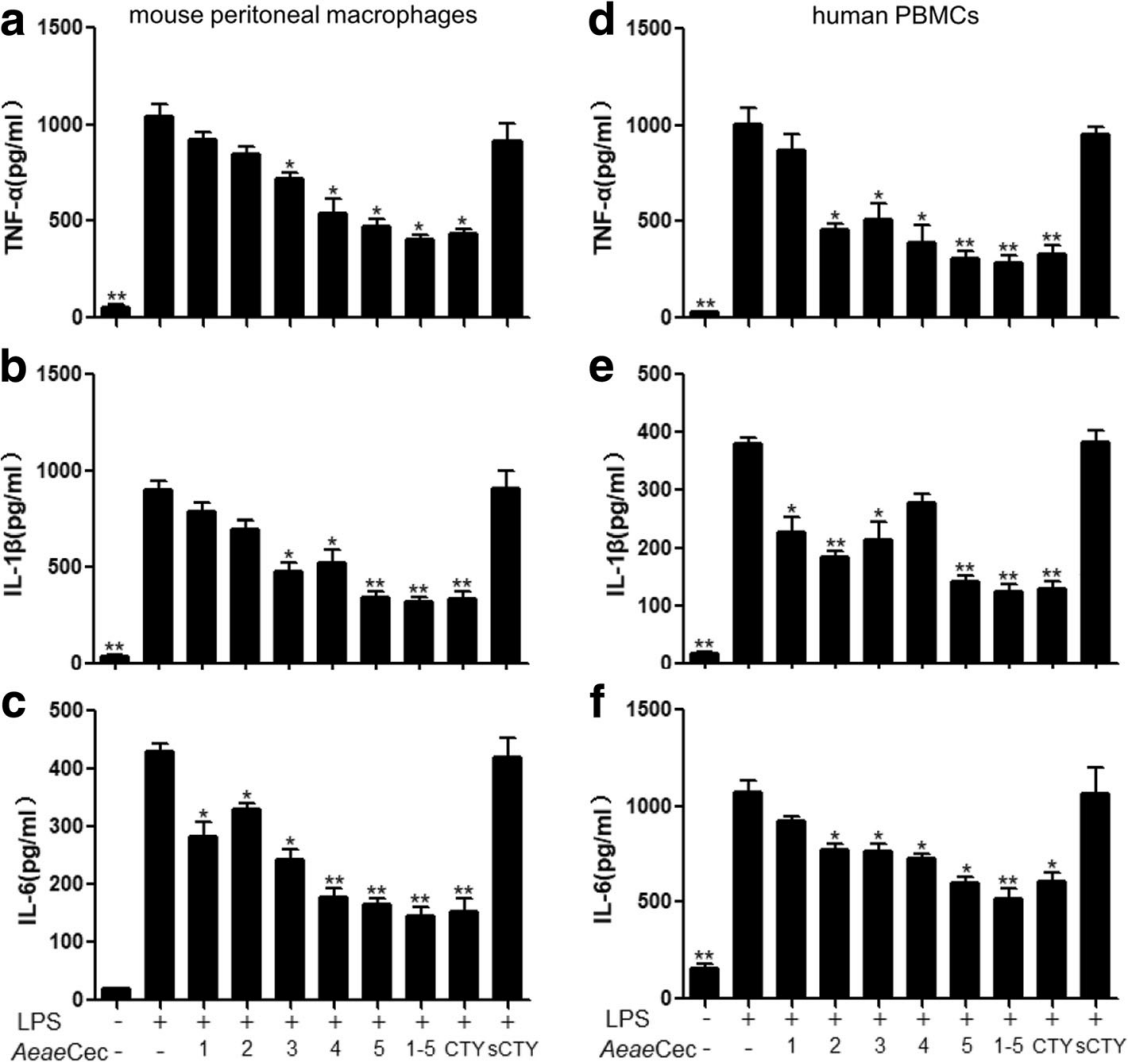
Inhibitory effects of individual and co-treatment of *Aedes aegypti* cecropins on LPS-induced pro-inflammatory cytokines production in mouse peritoneal macrophages and human PBMCs. Inhibitory effects of *Aeae*Cec1-5 on LPS-stimulated inflammatory response were analyzed by ELISA assay. Production of TNF-α, IL-1β andIL-6 in peritoneal macrophages (**a-c**) and in human PBMCs (**d-f**) were analyzed 6 h after LPS (100 ng/ml) incubation in presence of PBS, individual *Aeae*Cec peptides (5 μM), *Aeae*Cec1-5 mixture (1 μM each), cecropin-TY1 (CTY, 5 μM) and scrambled cecropin-TY1 (sCTY, 5 μM) as control peptide [25]. Data are presented as mean ± SEM from three independent experiments. *P < 0.05, **P < 0.01

To further elucidate the *in vitro* anti-inflammatory effect of *Aeae*Cec1-5 on macrophages and monocytes, we analyzed the effect of *Aeae*Cec1-5 on LPS-induced pro-inflammatory cytokine production in mouse peritoneal macrophages and human PBMCs. As shown in Fig. 2a-c, LPS (100 ng/ml) significantly induced high levels of TNF-α, IL-1β and IL-6 production in mouse macrophages, and 5 μM of individual*Aeae*Cec1-5could inhibit the production of the three cytokines with different inhibitory efficiencies. Among the five analogues, *Aeae*Cec5 had the strongest inhibitory effects on pro-inflammatory cytokine production, which reduced TNF-α production by 57.7% (*t*_(4)_ = 8.002, *P* = 0.0013, Fig. 2a), IL-1β production by 63.9% (*t*_(4)_ = 9.81, *P* = 0.0006, Fig. 2b), and IL-6 production by 64.4% (*t*_(4)_ = 12.01, *P* = 0.0003, Fig. 2c). Similar results were observed in LPS-stimulated human PMBCs, in which *Aeae*Cec1-5 showed inhibitory effects on LPS-induced pro-inflammatory cytokine production (Fig. 2d-f). Collectively, *Aeae*Cec5 was the most potent among the five peptides for attenuating TNF-α production by 71.6% (*t*_(4)_ = 8.977, *P* = 0.0009, Fig. 2d), IL-1β production by 65.9% (*t*_(4)_ = 5.437, *P* = 0.0056, Fig. 2e), and IL-6 production by 51.6% (*t*_(4)_ = 7.821, *P* = 0.0014, Fig. 2f). Incubation of *Aeae*Cec1-5 mixture (1 μM each) enhanced the individual-*Aeae*Cecs-mediated inhibitory effect on LPS-induced production of pro-inflammatory cytokines. *Aeae*Cec1-5 mixture incubation reduced TNF-α, IL-1β and IL-6 production by 64.5% (*t*_(4)_ = 9.903, *P* = 0.0006, Fig. 2a), 66.9% (*t*_(4)_ = 11.32, *P* = 0.0003, Fig. 2b) and 69.0% (*t*_(4)_ = 11.98, *P* = 0.0003, Fig. 2c) in mouse macrophages, and 73.9% (*t*_(4)_ = 9.547, *P* = 0.0007, Fig. 2d), 70.8% (*t*_(4)_ = 7.996, *P* = 0.0013, Fig. 2e) and 60.9% (*t*_(4)_ = 7.384, *P* = 0.0018, Fig. 2f) in human PBMCs, respectively.

### *Aeae*Cec1-5 showed low toxicity against mammalian cells

To determine the cytotoxicity of *Aeae*Cec1-5 against mammalian cells, Vero E6 cells, mouse peritoneal macrophages and human PBMCs were exposed to different peptide concentrations. As shown in Additional file 2: Figure S1, *Aeae*Cec1-4 incubation resulted in 100% cell viability at concentration up to 50 μM. At a concentration below 6.25 μM, *Aeae*Cec5 exhibited no cytotoxicity toward all tested cells, and showed minimal cytotoxicity at 12.5 μM towards mouse macrophages (9.5%) and PBMCs (8.9%). At a concentration of 50 μM, *Aeae*Cec5 caused 28.9 and 17.5% cell death toward mouse macrophages and human PBMCs, respectively, but no cytoxicity against Vero E6 cells. In addition, the co-treatment of *Aeae*Cec1-5 (1 μM each) did not induce any cell death toward all tested cells (data not shown). Thus, *Aeae*Cec1-5 showed efficiently anti-inflammatory activities with low toxicity against mammalian cells.

### *Aeae*Cec1-5 inhibited the activation of LPS-induced MAPKs and NF-κB signaling pathways

**Fig. 3:**
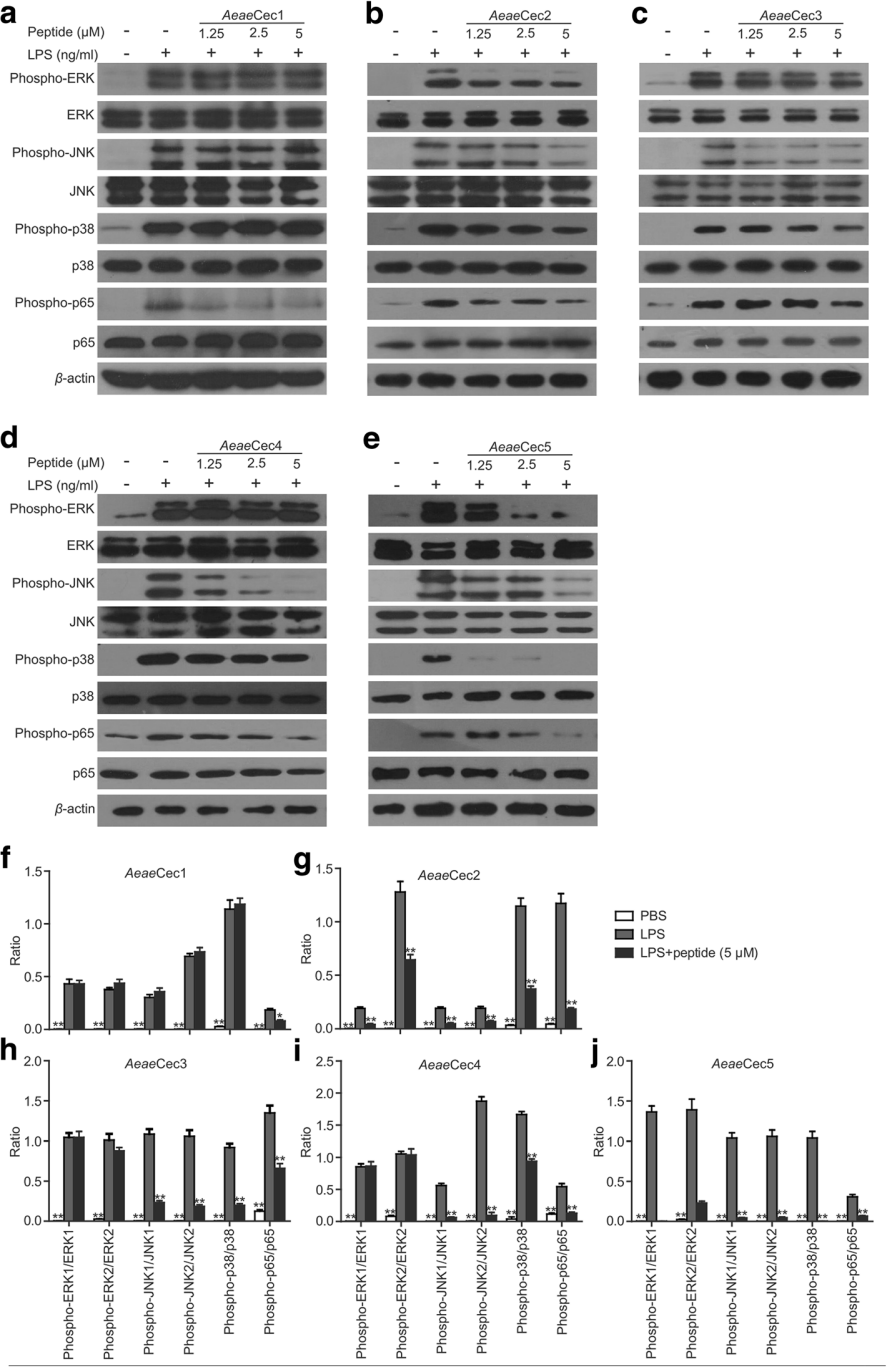
*Aedes aegypti* cecropins restrained the activation of MAPKs and NF-κB signaling pathways in LPS-stimulated macrophages. **a-e** Effects of *Aeae*Cec peptides on phosphorylation of ERK, JNK, p38, and NF-κB p65 induced by LPS. Mouse peritoneal macrophages were stimulated with LPS (100 ng/ml) for 30 min in the presence of 1.25 μM to 5 μM of individual *Aeae*Cec1 (**a**), *Aeae*Cec2 (**b**), *Aeae*Cec3 (**c**), *Aeae*Cec4 (**d**) and *Aeae*Cec5(**e**) peptide. Total and phosphorylation of ERK, JNK, p38 and NF-κB p65 were detected by western blot. **f-j** The ratios of phosphorylated-ERK, JNK, p38, NF-κB p65 to total ERK, JNK, p38, NF-κB p65 after *Aeae*Cec peptides treatment were determined, respectively. Band densities were analyzed using Quantity One software (Bio-Rad, Richmond, CA, USA). Data are presented as mean ± SEM from three independent experiments. *P < 0.05, **P < 0.01

Recognition of LPS by Toll like receptor 4 (TLR 4) initiates signaling pathways involving activation of MAPKs and NF-κB, which play critical roles in the upregulation of pro-inflammatory factors. To understand the anti-inflammatory mechanisms of *Aeae*Cec1-5, their effects on LPS-induced activation of MAPKs and NF-κB signaling pathways were detected. In mouse peritoneal macrophages, we detected significantly enhanced expression levels of phospho-JNK, ERK, p38 and NF-κB p65 after LPS (100 ng/ml) stimulation, which was selectively decreased by the treatment of *Aeae*Cec1-5 with different efficiency. This inhibitory effect occurred in a dose-dependent manner (Fig. 3). At a concentration of 5 μM, *Aeae*Cec1 only moderately reduced phosphorylation of NF-κB p65 by 56.9% (*t*_(4)_ = 6.29, *P* = 0.0033, Fig. 3a, f). *Aeae*Cec2 reduced phosphorylation of all kinases and transcriptional factor, with efficiencies from 49.2 to 84.1% (phospho-ERK1: *t*_(4)_ = 9.795, *P* = 0.0006; phospho-ERK2: *t*_(4)_ = 5.7656, *P* = 0.0045; phospho-JNK1: *t*_(4)_ = 9.042, *P* = 0.0008; phospho-JNK2: *t*_(4)_ = 6.886, *P* = 0.0023; phospho-p38: *t*_(4)_ = 9.689, *P* = 0.0006; phospho-NF-κB p65: *t*_(4)_ = 10.71, *P* = 0.0004; Fig. 3b, h). Both *Aeae*Cec3 and *Aeae*Cec4 significantly inhibited phosphorylation of JNK1 (*Aeae*Cec3: *t*_(4)_ = 12.85, *P* = 0.0002; *Aeae*Cec4: *t*_(4)_ = 14.03, *P* = 0.0001), JNK2 (*Aeae*Cec3: *t*_(4)_ = 10.88, *P* = 0.0004; *Aeae*Cec4: *t*_(4)_ = 12.67, *P* = 0.0002), p38 (*Aeae*Cec3: *t*_(4)_ = 13.67, *P* = 0.0002; *Aeae*Cec4: *t*_(4)_ = 12.41, *P* = 0.0002) and NF-κB p65 (*t*_(4)_ = 6.262, *P* = 0.0033; *Aeae*Cec4: *t*_(4)_ = 7.85, *P* = 0.0014) but not that of ERK1 and ERK2 (Fig. 3c, I and 3d, j). Among all the *Aeae*Cec peptides, *Aeae*Cec5 showed the strongest restriction on activation of the MAPKs and NF-κB signaling. Phosphorylation of JNK, ERK, p38 and NF-κB p65 were most efficiently inhibited by *Aeae*Cec5 treatment, especially that of ERK1, JNK1, and p38 which was attenuated by more than 90% (phospho-ERK1: *t*_(4)_ = 7.862, *P* = 0.0014; phospho-ERK2: *t*_(4)_ = 8.592, *P* = 0.001; phospho-JNK1: *t*_(4)_ = 15.21, *P* = 0.0001; phospho-JNK2: *t*_(4)_ = 12.60, *P* = 0.0002; phospho-p38: *t*_(4)_ = 12.76, *P* = 0.0002; phospho-NF-κB p65: *t*_(4)_ = 8.578, *P* = 0.001; Fig. 3e, k). These results indicated that the inhibitory effect of *Aeae*Cec1-5 on LPS-induced inflammation involves the effective downregulation of MAPKs and NF-κB signaling, with *Aeae*Cec5 representing the most potent peptide.

### *Aeae*Cec1-5 neutralized LPS

**Fig. 4:**
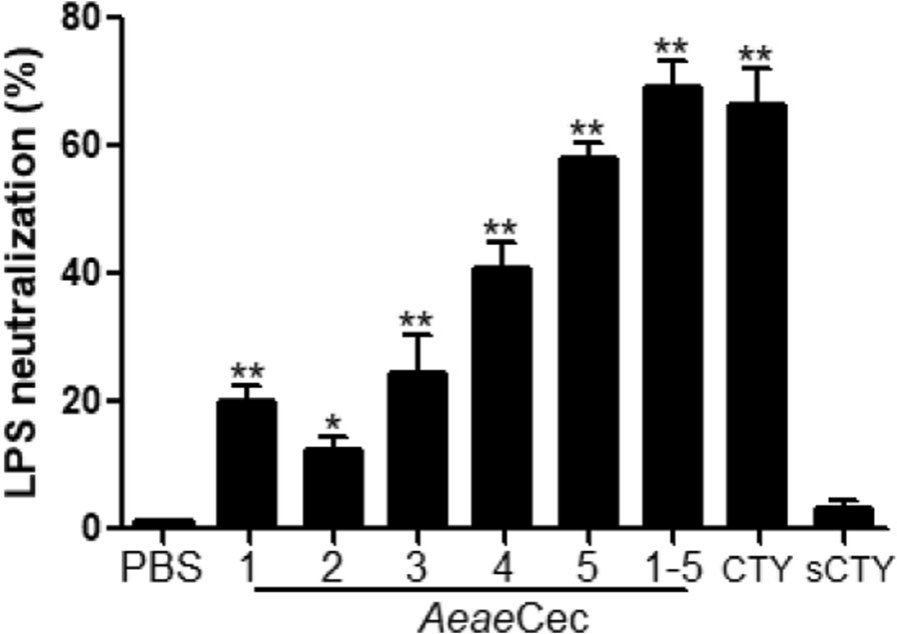
*Aedes aegypti* cecropins neutralized LPS. *E. coli* LPS (5 endotoxin units/ml) was incubated with 20 μM of individual *Aeae*Cec1-5 or their mixtures (4 μM each), vehicle (PBS), cecropin-TY1(CTY) or scrambled cecropin-TY1 (sCTY) as control peptide [25] at 37°C. The LPS-neutralization activity was evaluated using an endotoxin quantification kit. Data are presented as mean ± SEM from three independent experiments. *P < 0.05, **P < 0.01

Since cecropins are positively charged, they are anticipated to bind to and neutralize LPS. We therefore used an endotoxin quantitation kit to analyze the neutralization of *Aeae*Cec1-5 (20 μM) to *E. coli* LPS (5 endotoxin units/ml) using the chromogenic LAL assay. We determined that all *Aeae*Cec peptides significantly neutralized LPS, with *Aeae*Cec5 as the most efficient one showing 58.0% neutralization of LPS at 20 μM (*Aeae*Cec1: *t*_(4)_ = 8.484, *P* = 0.0011; *Aeae*Cec2: *t*_(4)_ = 6.165, *P* = 0.0035; *Aeae*Cec3: *t*_(4)_ = 3.648, *P* = 0.0218; *Aeae*Cec4: *t*_(4)_ = 9.827, *P* = 0.0006; *Aeae*Cec5: *t*_(4)_ = 11.74, *P* = 0.0003; Fig. 4).Treatment with *Aeae*Cec1-5 mixture (4 μM each) led to the best LPS-neutralization activity up to 68% (*t*_(4)_ = 11.30, *P* = 0.0003).

### *Aeae*Cec1-5 ameliorated inflammatory response and lung damage in LPS-challenged mice

**Fig. 5:**
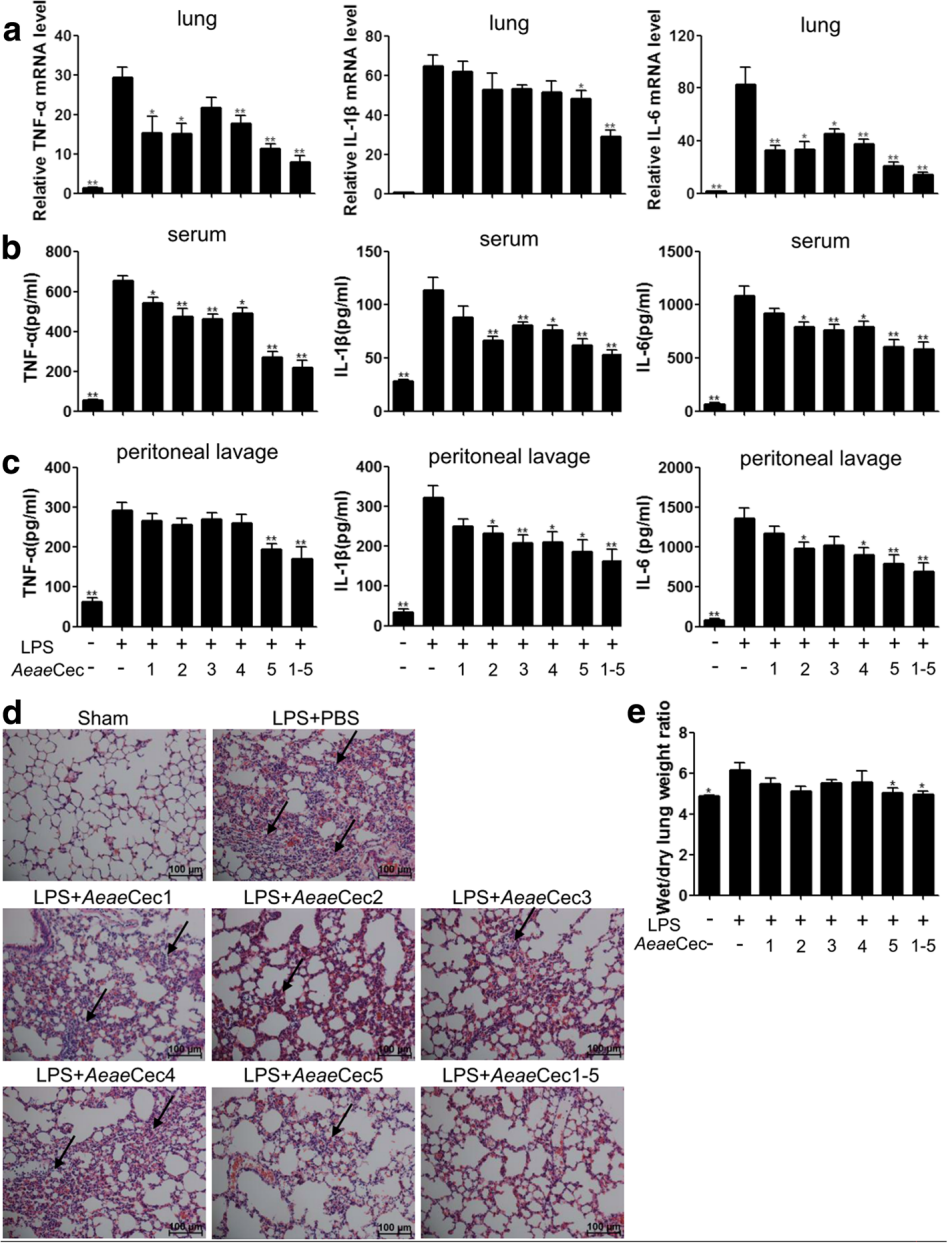
*Aedes aegypti* cecropins protected mice from LPS-induced endotoxin shock. C57BL/6 mice were i.p. injected with 10 mg/kg LPS (*E. coli* 0111:B4) and then treated with *Aeae*Cec peptides (10 mg/kg), *Aeae*Cec1-5 mixture (2 mg/kg each) or vehicle (PBS) for 4 h. **a-c** Expression of TNF-α, IL-1β and IL-6 in the lungs (**a**), serum (**b**) and peritoneal lavage (**c**) were detected by RT-PCR and ELISA assay. **d** Histopathological changes in the lungs. The tissue samples were stained with H&E. The arrows showed inflammatory infiltration. **e** The ratio of wet weight to dry weight of the lungs was evaluated. Data are presented as mean ± SEM from three independent experiments. *P < 0.05, **P < 0.01

To assess the anti-inflammatory effects of *Ae. aegypti* cecropins *in vivo*, C57BL/6 mice were intraperitoneally injected with 10 mg/kg LPS (from *E. coli* 0111:B4; Sigma-Aldrich) and then treated with *Aeae*Cec1-5 (10 mg/kg). As shown in Fig. 5a-c, *Aeae*Cec1-5 reduced the elevated mRNA levels of TNF-α and IL-6 in the lung, protein levels of TNF-α, IL-1β and IL-6 in serum, as well as protein levels of IL-1β and IL-6 in peritoneal lavage otherwise seen following LPS administration. Among the five cecropins, *Aeae*Cec5 treatment showed the best therapeutic effect, where the serum TNF-α, IL-1β and IL-6 levels were 270 (*t*_(17)_ = 9.428, *P* < 0.0001), 62 (*t*_(17)_ = 3.61, *P* = 0.0022) and 608 pg/ml (*t*_(17)_ = 4.341, *P* = 0.0004) with *Aeae*Cec5 treatment, respectively, compared with 654, 114 and 1087 pg/ml in serum induced with LPS only (Fig. 5b). The *Aeae*Cec1-5 mixture administration (2 mg/kg each) generated the best anti-inflammatory effect in the lung (*t*_(16)_ = 10.73, *P* < 0.0001), serum (*t*_(16)_ = 4.423, *P* = 0.0004) and peritoneal lavage (*t*_(16)_ = 4.609, *P* = 0.0003).

Consistent with these findings, lungs following *E. coli* 0111:B4 LPS-treated mice were significantly damaged with multi-inflammatory infiltration foci, but *Aeae*Cec5 administration significantly reversed this inflammatory damage (Fig. 5d), and the *Aeae*Cec1-5 mixture treatment further enhanced the protection. In addition to that, *Aeae*Cec5 and the *Aeae*Cec1-5 mixture also significantly reduced the wet/dry lung weight ratio to a level comparable to that seen in the naïve mice (*Aeae*Cec5: *t*_(8)_ = 2.538, *P* = 0.0348; *Aeae*Cec1-5: *t*_(8)_ = 2.846, *P* = 0.0216; Fig. 5e), indicating protection against LPS-induced lung edema. These data indicate that *Aeae*Cec5 is a strong inhibitor of endotoxin activity in C57BL/6 mice.

### *Aeae*Cec5 protected mice against *E. coli* and *P. aeruginosa* infection

**Fig. 6:**
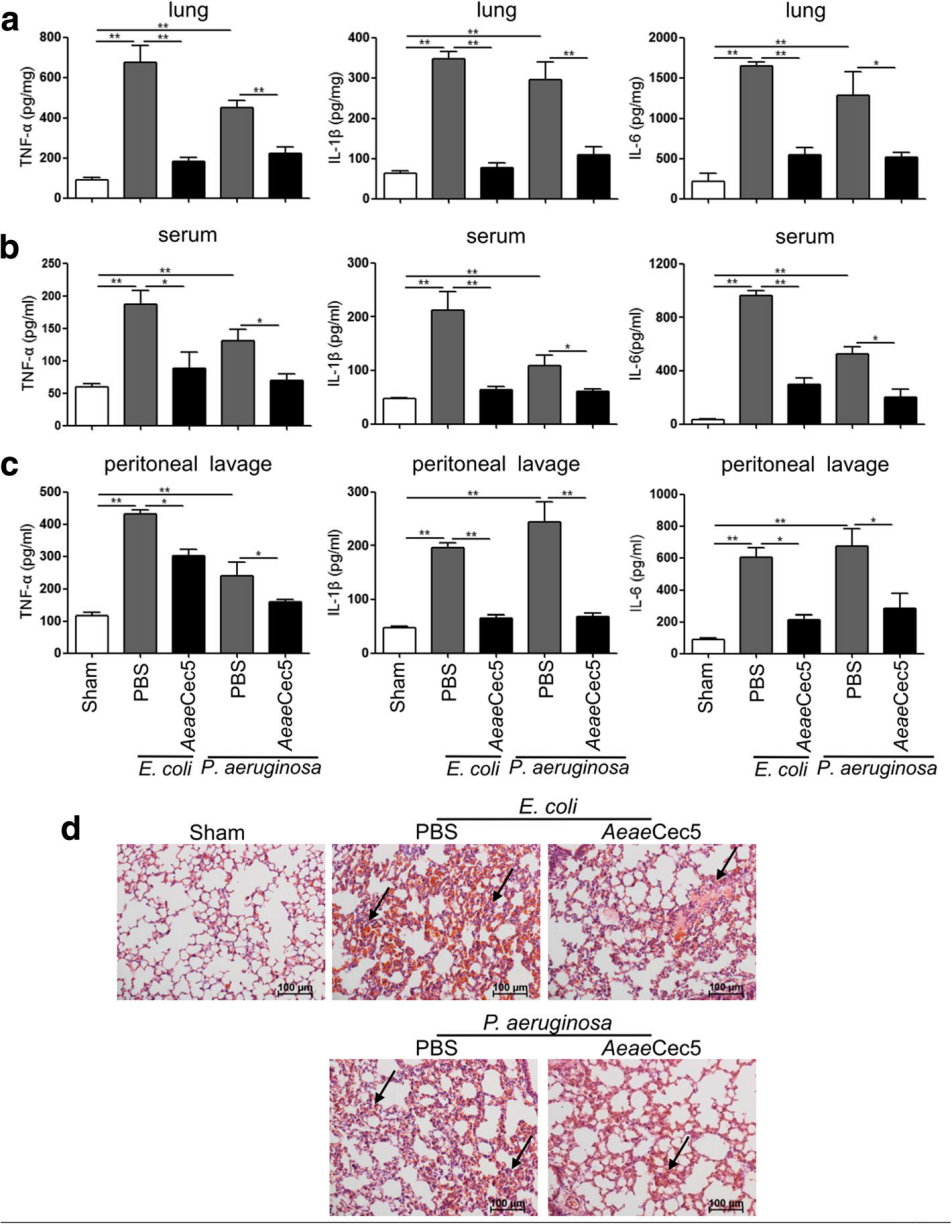
*Aeae*Cec5 offered anti-inflammatory protection against *E. coli* and *P. aeruginosa* infection. Mice were i.p. infected with *E. coli* or *P. aeruginosa* (2 × 10^7^ CFUs/mouse), and treated with one dose of *Aeae*Cec5 (10 mg/kg) or PBS 30 min post-infection. **a-c** 18 h after treatment, all the mice were analyzed for TNF-α, IL-1β and IL-6 production in the lung (**a**), blood (**b**) and peritoneal lavage (**c**) by ELISA assay. Data are presented as mean ± SEM from three independent experiments. **d** Representative H&E staining of the lung sections 18 h post-infection. The arrows showed typical injury sites. *Scale-bars*: 100 μm, *P < 0.05, **P < 0.01

To further test the protective activity of *Aeae*Cec5 in Gram-negative bacteria infection models, mice were intraperitoneally infected with two major infective bacteria in clinical infectious diseases, *E. coli* or *P. aeruginosa* (2×10^7^ CFUs/mouse), and then treated with *Aeae*Cec5 (10 mg/kg). After treatment for 18 h, a significant decrease in the levels of pro-inflammatory cytokines (TNF-α, IL-1β and IL-6) in the lung, serum, and peritoneal lavage was observed as compared to the levels of PBS-treated mice (Fig. 6a-c). Consistently, the pathology and the inflammatory injury in the lung were also significantly improved (Fig. 6d). The results indicated that *Aeae*Cec5 offered anti-inflammatoy protection against Gram-negative bacterial infection.

## Discussion

Mosquito vectors have been proven to produce a variety of physiologically active compounds that suppress their host’s hemostatic system and immune response to successfully get a blood meal [15, 25]. These physiologically active factors can be distinguished into two major classes, including anti-hemostatic and immunoregulatory substances [15, 24]. So far, much work has been done to study anti-hemostatic and immunoregulatory substances from mosquito vectors. This includes work on: (i) apyrases, which inhibited ADP-induced platelet aggregation and limited local blood coagulation for successful blood-feeding on their host [5, 13]; (ii) Sialokinin-I and Sialokinin-II, two mosquito neuropeptides of tachykinins, which had smooth muscle contracting activity [11]; (iii) salivary gland homogenates of *A. albimanus* which oxidized noradrenalin and effectively inhibited vasoconstrictive pathways [12]; (iv) AMPs, defensins, gambicins and cecropins comprising the three main antimicrobial peptide families, which were initially identified for their antimicrobial activity *in vitro* [15–19, 38]; (v) immunosuppressors, three pentapeptides derived from the C-terminal fragments of tachykinins with the general structure Phe-X-Gly-Leu-Met-NH2 (with Tyr, Val and Ile in X position) which showed potent immunosuppressive effects [45]; (vi) crude saliva of *Culex pipiens* and *Ae. aegypti* which modulated the cytokine production in C3H/HeJ mice [21]; (vii) cured saliva of *Ae. aegypti* which modulated murine lymphocyte function [46]; (viii) crude salivary gland extracts of *Ae. aegypti* which inhibited the production of TNF-α in tumour cell-stimulated mast cells [23]; and (ix) crude salivary gland extract of *Ae. aegypti* which modulated murine cellular and host immune responses [8, 22]. Collectively, several specific anticoagulant compounds have been well characterized from mosquitoes, and the immunomodulatory effects of crude saliva or salivary gland extract were described in mosquitoes. However, the specific anti-inflammatory compound from mosquito vectors is currently unknown.

In this study, we characterized *Aeae*Cec1-5 as anti-inflammatory AMPs in *Ae. aegypti*. We confirmed the anti-inflammatory activities of *Aeae*Cec1-5 against LPS-induced NO and pro-inflammatory cytokines production and the co-treatment of *Aeae*Cec1-5 generated the best anti-inflammatory effects. Among the five cecropins, *Aeae*Cec5 displayed the strongest anti-inflammatory activity induced by LPS *in vitro* and *in vivo* with low toxicity. Treatment with *Aeae*Cec5 in *E. coli* or *P. aeruginosa* infected mice also decreased the pro-inflammatory cytokines production and lung damage. *Aeae*Cec1-5 might modulate both cellular and host inflammatory response by reducing the production of inflammatory mediators from monocytes/macrophages at the bite site. A reduction of inflammatory mediator release may be beneficial for the feeding of *Ae. aegypti* by reducing immediate inflammatory response at the feeding site, suggesting that cecropins of *Ae. aegypti* might be the specific components that suppressed the inflammatory response of the mammalian host during feeding.

Mosquitoes have many chances to imbibe various microorganisms during feeding. Cecropins in mosquitoes were initially identified as antimicrobial compounds *in vitro* [15, 17] which may facilitate the killing of microorganisms in blood meals, and protect them from pathogenic infection during feeding. Herein, we characterized the anti-inflammatory effects of mosquito cecropins (*Aeae*Cec1-5) *in vivo*. Of the five *Ae. aegypti* cecropins, *Aeae*Cec5 showed the best protective effect against LPS-stimulated inflammatory response both *in vitro* and *in vivo*. LPS, also known as endotoxin, comprises the main components of the cell wall of Gram-negative bacteria. Gram-negative bacteria infection directly leads to the release of LPS that triggers the excessive production of systemic pro-inflammatory cytokines and NO, which is called cytokine storm, and ultimately results in sepsis [47, 48]. *Aeae*Cec5 was chosen to investigate the anti-inflammatory effects caused by Gram-positive bacteria infection in mice. The *in vivo* anti-inflammatory analyses of *Aeae*Cec5 induced by bacterial infection illustrated that *Aeae*Cec5 significantly ameliorated lung injury and pro-inflammatory cytokines production in mice after infected with Gram-negative bacteria *E. coli* and *P. aeruginosa*, which are two major infective bacteria in clinical infectious diseases (Fig. 6). Besides anti-inflammatory activity, *Aeae*Cec5 also showed strong antimicrobial activity against the four tested *E. coli* strains *in vitro* with minimal inhibitory concentrations ranging from 1.17 to 2.34 μg/ml (Additional file 1: Table S3), and bacterial load in peritoneal lavage of *E. coli*-infected mice was significantly reduced (appropriately 85.6%) after treatment of *Aeae*Cec5 (10 mg/kg) as compared to PBS treatment (Additional file 3: Figure S2). As described above, such defensive peptides from mosquito vectors were initially identified for their antimicrobial activity *in vitro* [15, 17]. In addition to antimicrobial activity, perhaps anti-inflammatory activity of these molecules like *Aeae*Cec5 is another strategy of *Ae. aegypti* to suppress the inflammatory response of mammalian host. As a result of co-evolution, cecropins are possibly the specific components that *Ae. aegypti* has developed to protect mammalian hosts from pathogen infection and pathogen-induced inflammatory response at the bite site during feeding. In our previous work, two cecropins from blood-feeding insects, the horsefly and black fly, showed anti-inflammatory properties similar to cecropins from the blood-feeding arthropod *Ae. aegypti* [7, 25], implying that cecropins might be common anti-inflammatory agents for blood-feeding.

As described previously, LPS is an agonist that target TLR 4 and subsequently activate the inflammatory pathways [26, 27]. The effects of *Aeae*Cec1-5 on LPS-activated inflammatory pathways were investigated to address the anti-inflammatory mechanisms (Fig. 3). In general, although *Aeae*Cec1-5 showed different inhibitory efficiency on the activation of inflammatory signal pathways, *Aeae*Cec1-5 selectively inhibited the activation (phosphorylation) of MAPKs and/or NF-κB signals in LPS-stimulated mouse macrophages. The results implied that cecropins of *Ae. aegypti* exerted their anti-inflammatory activities by blocking the activation (phosphorylation) of MAPKs and NF-κB signaling pathways, which in turn ameliorated the inflammatory response induced by LPS both *in vitro* and *in vivo*. In addition, *Aeae*Cec1-5 showed different effects on the inhibition of the phosphorylation of ERK, JNK, p38 and NF-κB p65, suggesting that this may be the molecular basis for the best anti-inflammatory effects of the co-treatment of *Aeae*Cec1-5. To understand the potential interactions between *Aeae*Cec peptides, the percent of inhibition of mixtures of two, three, four, and five peptides in LPS-stimulated NO production in mouse peritoneal macrophages were investigated systematically. Among these different sets of mixture of peptides, the best protective effect in mixtures of two, three, four, or five peptides was only generated in the presence of *Aeae*Cec5 (Additional file 1: Table S4). The interactive effects between any two peptides of *Aeae*Cec1-5 on inhibition of NO production in LPS-stimulated mouse peritoneal macrophages were assayed using chequerboard assays. The results indicated that additive effects were observed in mixtures of *Aeae*Cec1 and *Aeae*Cec5, *Aeae*Cec2 and *Aeae*Cec5, *Aeae*Cec3 and *Aeae*Cec5, and *Aeae*Cec4 and *Aeae*Cec5 (Additional file 1: Table S5). Insect-derived cecropins adopt a random coil structure in aqueous solution but convert to an α-helical structure in the hydrophobic environments by forming an N-terminal helix region and a C-terminal helix region linked by hinge [35]. Upon the interaction with LPS micelles (hydrophobic environments), *Aeae*Cec1-5 possibly possess an N-terminal amphipathic α-helix and a more hydrophobic C-terminal α-helix connected by a hinge region as *Aeae*Cec4 (Aedesin) detected by nuclear magnetic resonance spectroscopy. These helical structures in such peptides are critical for their neutralization of endotixion (LPS) and anti-inflammatory activities [25, 27]. In addition to the helical structures, *Aeae*Cec5 has a tryptophan residue (Trp2) in the N-terminal helix, which is missing from *Aeae* Cec1, 2, 3 and 4. As described previously, aromatic residues like Trp in the N-terminus are required for the interaction of cecropin peptides with LPS [25, 49], and *Aeae*Cec5 did exhibit the strongest LPS-neutralizing activity and anti-inflammatory activities among these five *Aeae*Cec peptides. Collectively, we suggest that these structural properties may contribute to the additive effects generated by *Aeae*Cec5 with other *Aeae*Cecs. To test this hypothesis, we substituted theTrp2 with Ala2, and the additive effects generated by *Aeae*Cec5(2W→2A) with other *Aeae*Cec peptides were absent (Additional file 1: Table S6). Additionally, another anti-inflammatory mechanism was addressed by evaluation of the LPS-neutralization activity of cecropins of *Ae. aegypti*. As shown in Fig. 4, *Aeae*Cec1-5 exhibited LPS-neutralization activity, suggesting that LPS-neutralization activity of *Ae. aegypti* cecropins at least partly comprised their anti-inflammatory mechanism. Some anti-inflammatory peptides, including cathelididins and cecropins, were also known to have LPS-neutralization activity [7, 25–27, 33, 34, 50]. Such LPS-neutralization activity comprised a critical mechanism of their anti-inflammatory effects.

## Conclusions

In summary, cecropins were characterized as specific anti-inflammatory compounds in *Ae. aegypti* with low cytotoxicity. Among the five cecropins, *Aeae*Cec5 had the strongest anti-inflammatory activity *in vitro* and *in vivo* induced by LPS and could offer anti-inflammatory protection against Gram-negative bacteria infection. The inhibitory effect of *Aeae*Cec5 on LPS-induced inflammation involved the suppression of TNF-α, IL-1β and IL-6 expression by interfering with the MAPKs and NF-κB pathways as well as neutralizing LPS. These properties make *Aeae*Cec5 a promising peptide candidate for the therapy of sepsis and endotoxin shock caused by Gram-negative bacteria infection, and provide new clues to the molecular basis of anti-inflammatory activities in mosquito vectors.

## Additional files

Additional file 1: **Table S1.** Information and amino acid sequence of *Ae. aegypti* cecropins. **Table S2.** Primer sequences for qPCR of mice. **Table S3.** Antimicrobial activities of *Aeae*Cec peptides *in vitro*. **Table S4.** Percent of inhibition of individual or mixtures of peptides in LPS-stimulated NO production in mouse peritoneal macrophages. **Table S5.** Chequerboard assays of any two peptides of *Aeae*Cec1-5 in inhibition of NO production in LPS-stimulated mouse peritoneal macrophages. **Table S6.** Chequerboard assays of *Aeae*Cec5(2W→2A) and other *Aeae*Cec peptides in inhibition of NO production in LPS-stimulated mouse peritoneal macrophages. (DOCX 31 kb)

Additional file 2: **Figure S1.** Cytotoxicity of *Ae. aegypti* cecropins against mammalian cells. **a** Mice peritoneal macrophages, human PBMCs and Vero E6 cells were exposed to 50 μM *Aeae*Cec1-5, respectively. **b** Mice peritoneal macrophages and human PBMCs were exposed to series of two-fold *Aeae*Cec5 dilutions ranging from 3.125 to 50 μM, respectively. (TIF 161 kb)

Additional file 3: **Figure S2.** *Aeae*Cec5 significantly reduced the bacteria CFUs in peritoneal lavage of *E. coli*-infected mice. (TIF 61 kb)
