# Supplementary material for: Anti-inflammatory activities of Aedes aegypti cecropins and their protection against murine endotoxin shock
Source: Parasit Vectors. 2018 Aug 14;11:470. doi: 10.1186/s13071-018-3000-8 (PMC6092832; doi:10.1186/s13071-018-3000-8)
Supplement: Supplementary file 1 — Table S1. Information and amino acid sequence of Ae. aegypti cecropins. Table S2. Primer sequences for qPCR of mice. Table S3. Antimicrobial activities of AeaeCec peptides in vitro. Table S4. Percent of inhibition of individual or mixtures of peptides in LPS-stimulated NO production in mouse peritoneal macrophages. Table S5. Chequerboard assays of any two peptides of AeaeCec1-5 in inhibition of NO production in LPS-stimulated mouse peritoneal macrophages. Table S6. Chequerboard assays of AeaeCec5(2W→2A) and other AeaeCec peptides in inhibition of NO production in LPS-stimulated mouse peritoneal macrophages. (DOCX 31 kb) [file 13071_2018_3000_MOESM1_ESM.docx]

**Additional file 1**

**Table S1. Information and amino acid sequence of *A. aegypti* cecropins**

| Name^a^ | Orignal name | GeneBank No. | Amino acid sequence | origin |
| --- | --- | --- | --- | --- |
| *Aeae*Cec1 | cecropin A | AAF59831.1,  AAL85581.1 | GGLKKLGKKLEGAGKRVFNAAEKALPVVAGAKALRK | hemolymph |
| *Aeae*Cec2 | cecropin A | AAK73081.1, P82592.2, AAL85582.1 | GGLKKLGKKLEGAGKRVFNAAEKALPVVAGAKAL-NH_2_ | not described |
| *Aeae*Cec3 | cecropin B1 | AHX25874.1 | GGLKKLGKKLEGVGKRVFKASEKALPVVTGYKAI-NH_2_ | not described |
| *Aeae*Cec4 | AAEL000598-PA,  Aedesin | XP_001649181.2 | GGLKKLGKKLEGAGKRVFKASEKALPVVVGIKAI-NH_2_ | salivary gland |
| *Aeae*Cec5 | cecropin N,AAEL000621-PA | AHH41648.1, EAT48339.1, | RWKFGKKLEKVGKNVFNAAKKALPVVAGYKAL-NH_2_ | not described |

^a^Short name used in the present work.

**Table S2. Primer sequences for qPCR of mice**

| Primer | Forward | Reverse |
| --- | --- | --- |
| TNF-α | CGGTGCCTATGTCTCAGCCT | GAGGGTCTGGGCCATAGAAC |
| IL-1β | ATGGCAACTGTTCCTGAACTC | GCCCATACTTTAGGAAGACA |
| IL-6 | AGTTGCCTTCTTGGGACTGA | TCCACGATTTCCCAGAGAAC |
| iNOS | CTGCAGCACTTGGATCAGGAACCTG | GGAGTAGCCTGTGTGCACCTGGAA |
| GAPDH | GTGAAGGTCGGTGTGAACGGATT | GGAGATGATGACCCTTTTGGCTC |

**Table S3. Antimicrobial activities of *Aeae*Cec peptides *in vitro***

| Microorganisms | MIC (μg/ml) | | | | |
| --- | --- | --- | --- | --- | --- |
|  | *Aeae*Cec1 | *Aeae*Cec2 | *Aeae*Cec3 | *Aeae*Cec4 | *Aeae*Cec5 |
| *E. coli* ATCC 25922 | 4.69 | 4.69 | 4.69 | 4.69 | 1.17 |
| *E. coli* ATCC 35218 | 4.69 | 4.69 | 4.69 | 4.69 | 2.34 |
| *E. coli* 13A10022 (CI) | 9.38 | 9.38 | 9.38 | 9.38 | 2.34 |
| *E. coli* 08A852 (CI) | 4.69 | 4.69 | 4.69 | 4.69 | 1.17 |

MIC: minimal inhibitory concentration. These concentrations represent mean values of three independent experiments performed in duplicates. CI: clinically isolated strain.

**Table S4. Percent of inhibition of individual or mixtures of peptides in LPS-stimulated NO production in mouse peritoneal macrophages**^a^

| Peptide alone or in combination | Percent of inhibition (%) |
| --- | --- |
| *Aeae*Cec1 | 57.6±6.3 |
| *Aeae*Cec2 | 57.9±5.7 |
| *Aeae*Cec3 | 52.2±6.9 |
| *Aeae*Cec4 | 48.0±5.4 |
| *Aeae*Cec5 | 78.1±7.2 |
| *Aeae*Cec1,2 | 55.2±8.1 |
| *Aeae*Cec1,3 | 54.4±5.9 |
| *Aeae*Cec1,4 | 51.6±6.2 |
| *Aeae*Cec1,5 | 79.7±8.6 |
| *Aeae*Cec2,3 | 53.1±6.4 |
| *Aeae*Cec2,4 | 51.5±7.8 |
| *Aeae*Cec2,5 | 83.6±8.5 |
| *Aeae*Cec3,4 | 49.2±5.5 |
| *Aeae*Cec3,5 | 81.4±9.2 |
| *Aeae*Cec4,5 | 75.5±8.7 |
| *Aeae*Cec1,2,3 | 54.3±7.4 |
| *Aeae*Cec1,2,4 | 52.2±5.6 |
| *Aeae*Cec1,2,5 | 80.6±6.6 |
| *Aeae*Cec1,3,4 | 52.1±4.9 |
| *Aeae*Cec1,3,5 | 78.6±8.3 |
| *Aeae*Cec1,4,5 | 79.5±7.6 |
| *Aeae*Cec2,3,4 | 52.4±5.8 |
| *Aeae*Cec2,3,5 | 78.4±6.7 |
| *Aeae*Cec2,4,5 | 77.1±6.4 |
| *Aeae*Cec3,4,5 | 76.7±7.8 |
| *Aeae*Cec1,2,3,4 | 50.7±6.1 |
| *Aeae*Cec1,2,3,5 | 81.3±7.8 |
| *Aeae*Cec1,2,4,5 | 77.6±8.2 |
| *Aeae*Cec1,3,4,5 | 78.6±7.5 |
| *Aeae*Cec2,3,4,5 | 79.3±6.9 |
| *Aeae*Cec1,2,3,4,5 | 84.2±9.7 |

^a^Mouse macrophages were incubated at 37˚C with *E. coli* LPS (100 ng/ml) in the presence or absenceof each *Aeae*Cec peptide (5 μM), mixture of two (5/2 μM each), three (5/3 μM each), four (5/4 μM each), or five peptides (5/5 μM each). After incubation for 24 h, the culture supernatants of mouse macrophages were detected for the NO production (nitrite accumulation) using Griess reagent. Data were calculated from three independent experiments and presented as mean ± SEM.

**Table S5. Chequerboard assays of any two peptides of *Aeae*Cec1~5 in inhibition of NO production in LPS-stimulated mouse peritoneal macrophages**^a^

| Peptide combination | IC50 (μg/ml) | | FIC | FICI |
| --- | --- | --- | --- | --- |
|  | Alone | Combination |  |  |
| *Aeae*Cec1 | 4.18±0.44 | 2.16±0.29 | 0.52 | 1.02 |
| *Aeae*Cec2 | 4.16±0.54 | 2.09±0.18 | 0.50 |  |
| *Aeae*Cec1 | 4.18±0.44 | 2.22±0.20 | 0.53 | 1.02 |
| *Aeae*Cec3 | 4.82±0.63 | 2.38±0.27 | 0.49 |  |
| *Aeae*Cec1 | 4.18±0.44 | 2.24±0.16 | 0.54 | 1.04 |
| *Aeae*Cec4 | 5.23±0.67 | 2.59±0.31 | 0.50 |  |
| *Aeae*Cec1 | 4.18±0.44 | 1.76±0.16 | 0.42 | 0.86 |
| *Aeae*Cec5 | 2.66±0.40 | 1.18±0.14 | 0.44 |  |
| *Aeae*Cec2 | 4.16±0.54 | 2.04±0.12 | 0.49 | 1.01 |
| *Aeae*Cec3 | 4.82±0.63 | 2.51±0.26 | 0.52 |  |
| *Aeae*Cec2 | 4.16±0.54 | 2.20±0.17 | 0.53 | 1.02 |
| *Aeae*Cec4 | 5.23±0.67 | 2.56±0.12 | 0.49 |  |
| *Aeae*Cec2 | 4.16±0.54 | 1.94±0.21 | 0.47 | 0.89 |
| *Aeae*Cec5 | 2.66±0.40 | 1.12±0.13 | 0.42 |  |
| *Aeae*Cec3 | 4.82±0.63 | 2.40±0.15 | 0.50 | 1.02 |
| *Aeae*Cec4 | 5.23±0.67 | 2.67±0.22 | 0.52 |  |
| *Aeae*Cec3 | 4.21±0.58 | 1.98±0.19 | 0.47 | 0.94 |
| *Aeae*Cec5 | 2.66±0.40 | 1.24±0.11 | 0.47 |  |
| *Aeae*Cec4 | 5.23±0.67 | 2.24±0.18 | 0.43 | 0.89 |
| *Aeae*Cec5 | 2.66±0.40 | 1.22±.0.18 | 0.46 |  |

^a^Anti-inflammatory interactions between peptide 1 and peptide 2 of *Aeae*Cec peptides were determined by the chequerboard assay as described previously [1]. First, 2-fold serial dilutions of peptides were prepared. Next, peptides were dissolved in PBS and added to mouse macrophages to final concentrations of 5, 2.5, 1.25, 0.625, 0.3125 μMin each well of a 96-well plate in the presence of *E. coli* LPS (100 ng/ml). The same volume of PBS served as a control. After incubation at 37˚Cfor 24 h, the culture supernatants of mouse macrophages were detected for the NO production (nitrite accumulation) using Griess reagent. Half maximal inhibitory concentrations (IC_50_) were calculated. The fractional inhibitory concentration index (FICI) was calculated for each combination using this equation: FICI = FIC_A_+ FIC_B_, where FIC_A_= IC_50_ of peptide A in combination/IC_50_ of peptide A alone, and FIC_B_= IC_50_ of peptide B in combination/IC_50_ of peptide B alone. FICI of ≤ 0.5 was interpreted as synergy, 0.5 < FICI ≤ 1.0 as additive, 1.0 < FICI ≤ 4.0 as indifferent, and FICI > 4.0 as antagonism [2, 3]. Data were calculated from three independent experiments and presented as mean ± SEM.

**Table S6. Chequerboard assays of *Aeae*Cec5(2W→2A) and other *Aeae*Cec peptides in inhibition of NO production in LPS-stimulated mouse peritoneal macrophages**^a^

| Peptide combination | IC50 (μg/ml) | | FIC | FICI |
| --- | --- | --- | --- | --- |
|  | Alone | Combination |  |  |
| *Aeae*Cec5(2W→2A) | 4.81±0.58 | 2.92±0.26 | 0.61 | 1.08 |
| *Aeae*Cec1 | 4.18±0.44 | 1.98±0.14 | 0.47 |  |
| *Aeae*Cec5(2W→2A) | 4.81±0.58 | 2.74±0.22 | 0.57 | 1.07 |
| *Aeae*Cec2 | 4.16±0.54 | 2.06±0.24 | 0.50 |  |
| *Aeae*Cec5(2W→2A) | 4.81±0.58 | 2.59±0.16 | 0.54 | 1.05 |
| *Aeae*Cec3 | 4.82±0.63 | 2.47±0.19 | 0.51 |  |
| *Aeae*Cec5(2W→2A) | 4.81±0.58 | 2.68±0.21 | 0.56 | 1.09 |
| *Aeae*Cec4 | 5.23±0.67 | 2.77±0.17 | 0.53 |  |

^a^Anti-inflammatory interactions between peptide 1 and peptide 2 were determined by the chequerboard assay as described previously [1]. First, 2-fold serial dilutions of peptides were prepared. Next, peptides were dissolved in PBS and added to mouse macrophages to final concentrations of 5, 2.5, 1.25, 0.625, 0.3125 μM in each well of a 96-well plate in the presence of *E. coli* LPS (100 ng/ml). The same volume of PBS served as a control. After incubation at 37˚Cfor 24 h, the culture supernatant of mouse macrophages were assayed for NO production (nitrite accumulation) using Griess reagent. Half maximal inhibitory concentrations (IC_50_) were calculated. The fractional inhibitory concentration index (FICI) was calculated for each combination using this equation: FICI = FIC_A_+ FIC_B_, where FIC_A_= IC_50_ of peptide A in combination/IC_50_ of peptide A alone, and FIC_B_= IC_50_ of peptide B in combination/IC_50_ of peptide B alone. FICI of ≤ 0.5 was interpreted as synergy, 0.5 < FICI ≤1.0 as additive, 1.0 < FICI ≤ 4.0 as indifferent, and FICI > 4.0 as antagonism [2, 3]. Data were calculated from three independent experiments and presented as mean ± SEM.

**References**

[1] Rand K, Houck H, Brown P, Bennett D. Reproducibility of the microdilutioncheckerboard method for antibiotic synergy. Antimicrob Agents Chemother1993;37:613e5.

[2] Jacobs DS, DeMott WR, Oxley DK. Jacobs &DeMott laboratory test handbookwith key word index. 5th ed. Hudson, OH: Lexi Comp; 2001.

[3] Eliopoulos GM, Moellering RC. Antibiotic combinations. 3rd ed. Baltimore,MD: The Williams & Wilkins Co.; 1991.
